# Supplementary material for: Scalable Scientific Interest Profiling Using Large Language Models
Source: ArXiv. 2025 Aug 19:arXiv:2508.15834v1. Preprint. [Version 1] (PMC12393255)
Supplement: Supplement 1 [file NIHPP2508.15834v1-supplement-1.pdf]

Supplementary materials

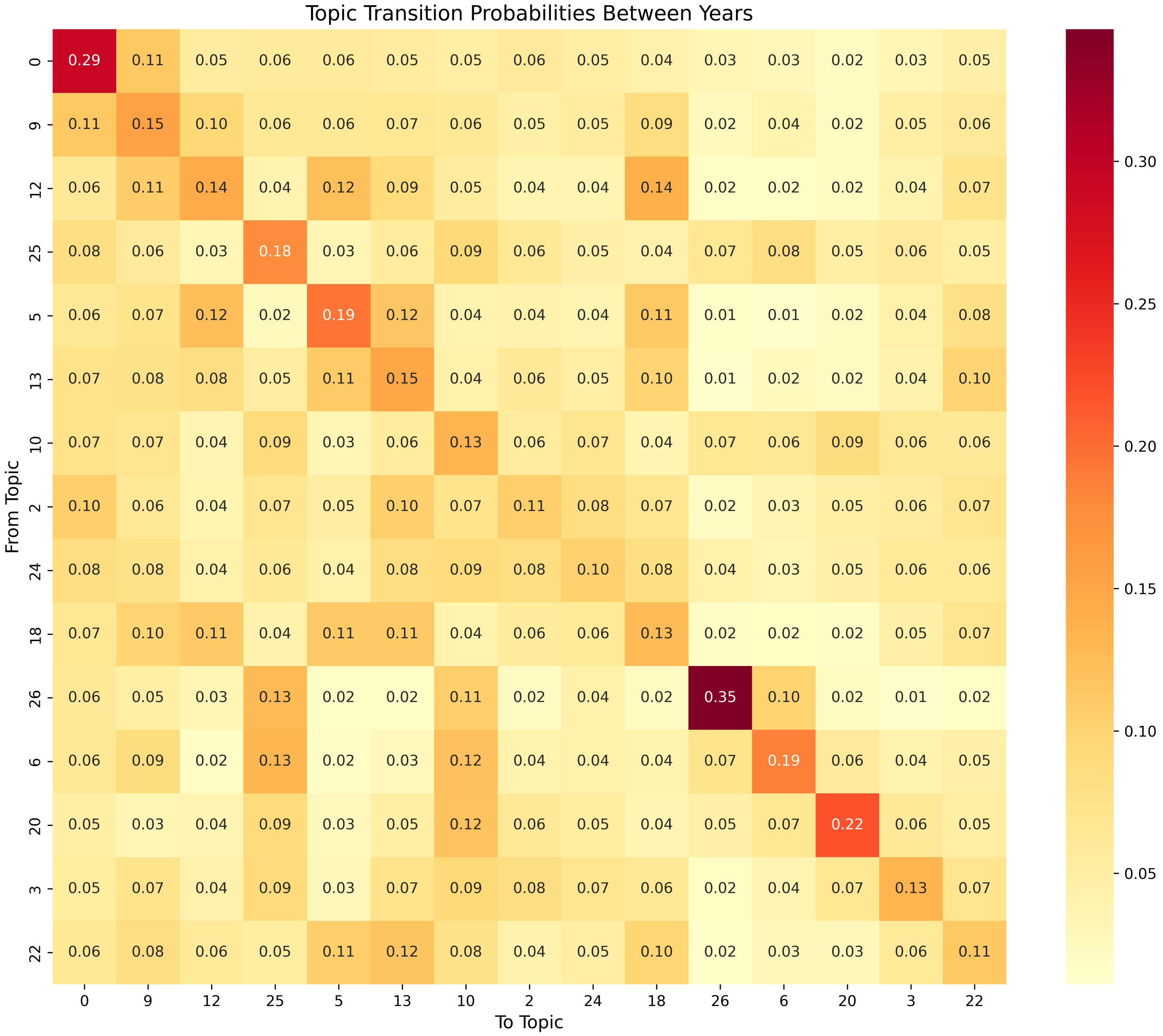

Supplementary Figure 1: Heatmap of researchers' topic transition probabilities between years.

Supplementary Table 1: Human evaluation results of self-written profiles.

Faculty	Overall impression	Factual Accuracy	Granularity	Readability	Comprehensiveness	Specificity	Conciseness	Identified as human
1	Excellent	Excellent	Detailed	Excellent	Excellent	Excellent	Good	TRUE
	Excellent	Excellent	Good granularity	Excellent	Excellent	Excellent	Excellent	TRUE
	Excellent	Excellent	Good granularity	Excellent	Excellent	Excellent	Good	FALSE
2	Excellent	Excellent	Good granularity	Excellent	Good	Excellent	Excellent	TRUE
	Excellent	Excellent	Good granularity	Excellent	Excellent	Excellent	Excellent	TRUE
	Excellent	Excellent	Good granularity	Excellent	Excellent	Excellent	Excellent	TRUE
3	Excellent	Excellent	Good granularity	Excellent	Excellent	Excellent	Excellent	TRUE
	Excellent	Excellent	Good granularity	Excellent	Excellent	Excellent	Excellent	TRUE
	Excellent	Excellent	Good granularity	Excellent	Excellent	Excellent	Excellent	TRUE
4	Excellent	Excellent	Good granularity	Excellent	Excellent	Excellent	Excellent	TRUE
	Excellent	Excellent	Good granularity	Excellent	Excellent	Excellent	Excellent	TRUE
	Excellent	Excellent	Good granularity	Excellent	Excellent	Excellent	Excellent	TRUE
5	Good	Excellent	Too detailed	Fair	Good	Fair	Fair	TRUE
	Excellent	Excellent	Good granularity	Excellent	Excellent	Excellent	Excellent	TRUE
	Excellent	Excellent	Detailed	Good	Excellent	Good	Fair	FALSE
6	Excellent	Excellent	Good granularity	Excellent	Excellent	Excellent	Excellent	TRUE
	Excellent	Excellent	Good granularity	Fair	Excellent	Excellent	Excellent	TRUE
	Excellent	Excellent	Good granularity	Good	Excellent	Excellent	Excellent	TRUE
7	Excellent	Excellent	Good granularity	Excellent	Excellent	Excellent	Excellent	TRUE
	Excellent	Excellent	Good granularity	Excellent	Excellent	Excellent	Excellent	TRUE
	Excellent	Excellent	Good granularity	Excellent	Excellent	Excellent	Excellent	TRUE
8	Excellent	Excellent	Good granularity	Excellent	Excellent	Excellent	Excellent	TRUE
	Excellent	Excellent	Good granularity	Excellent	Excellent	Excellent	Excellent	TRUE
	Excellent	Excellent	Good granularity	Excellent	Excellent	Excellent	Excellent	TRUE
9	Excellent	Excellent	Good granularity	Excellent	Excellent	Excellent	Excellent	TRUE
	Excellent	Excellent	Good granularity	Excellent	Excellent	Excellent	Excellent	TRUE
	Excellent	Excellent	Good granularity	Excellent	Excellent	Excellent	Excellent	TRUE
10	Excellent	Excellent	Good granularity	Excellent	Excellent	Excellent	Excellent	TRUE
	Excellent	Excellent	Good granularity	Excellent	Excellent	Excellent	Excellent	TRUE
	Excellent	Excellent	Good granularity	Excellent	Excellent	Excellent	Excellent	TRUE
11	Excellent	Excellent	Good granularity	Excellent	Excellent	Excellent	Excellent	TRUE
	Excellent	Excellent	Good granularity	Excellent	Excellent	Excellent	Excellent	TRUE
	Excellent	Excellent	Good granularity	Excellent	Excellent	Excellent	Excellent	TRUE
12	Excellent	Excellent	Good granularity	Excellent	Excellent	Excellent	Excellent	TRUE
	Excellent	Excellent	Good granularity	Excellent	Excellent	Excellent	Excellent	TRUE
	Excellent	Excellent	Good granularity	Excellent	Excellent	Excellent	Excellent	TRUE
13	Excellent	Excellent	Good granularity	Excellent	Excellent	Excellent	Excellent	TRUE
	Excellent	Excellent	Good granularity	Excellent	Excellent	Excellent	Excellent	TRUE
	Excellent	Excellent	Good granularity	Excellent	Excellent	Excellent	Excellent	TRUE
14	Excellent	Excellent	Good granularity	Excellent	Excellent	Excellent	Excellent	TRUE
	Excellent	Excellent	Good granularity	Excellent	Excellent	Excellent	Excellent	TRUE
	Excellent	Excellent	Good granularity	Excellent	Excellent	Excellent	Excellent	TRUE
15	Excellent	Excellent	Good granularity	Excellent	Excellent	Excellent	Excellent	TRUE
	Good	Excellent	General	Excellent	Excellent	Excellent	Excellent	TRUE
	Excellent	Excellent	Good granularity	Excellent	Excellent	Excellent	Excellent	TRUE
16	Excellent	Excellent	Good granularity	Excellent	Excellent	Excellent	Excellent	TRUE
	Excellent	Excellent	General	Excellent	Excellent	Excellent	Excellent	TRUE
	Excellent	Excellent	General	Excellent	Excellent	Excellent	Excellent	TRUE
17	Excellent	Excellent	Good granularity	Excellent	Excellent	Excellent	Excellent	TRUE
	Excellent	Excellent	Good granularity	Excellent	Excellent	Excellent	Excellent	TRUE
	Excellent	Excellent	Good granularity	Excellent	Excellent	Excellent	Excellent	TRUE
18	Excellent	Excellent	Good granularity	Excellent	Excellent	Excellent	Excellent	TRUE
	Good	Excellent	General	Excellent	Good	Excellent	Excellent	TRUE
	Excellent	Excellent	General	Excellent	Excellent	Excellent	Excellent	TRUE

Supplementary Table 2: Human evaluation results of MeSH term-based profiles.

Faculty	Overall impression	Factual Accuracy	Granularity	Readability	Comprehensiveness	Specificity	Conciseness	Identified as human
1	Poor	Poor	Good granularity	Good	Good	Very poor	Good	FALSE
	Fair	Poor	Too general	Good	Fair	Fair	Fair	FALSE
	Poor	Fair	General	Good	Fair	Poor	Good	FALSE
2	Good	Good	Detailed	Excellent	Excellent	Good	Good	FALSE
	Fair	Fair	General	Good	Fair	Fair	Poor	FALSE
	Good	Fair	Good granularity	Excellent	Good	Fair	Good	FALSE
3	Good	Fair	General	Good	Fair	Poor	Fair	FALSE
	Good	Good	General	Good	Good	Poor	Fair	FALSE
	Very poor	Very poor	Detailed	Excellent	Good	Very poor	Fair	FALSE
4	Good	Good	Good granularity	Excellent	Excellent	Good	Good	FALSE
	Good	Good	Good granularity	Excellent	Excellent	Good	Excellent	FALSE
	Fair	Excellent	Detailed	Poor	Good	Fair	Good	FALSE
5	Excellent	Excellent	Good granularity	Excellent	Good	Excellent	Excellent	TRUE
	Good	Good	Good granularity	Good	Fair	Fair	Excellent	FALSE
	Excellent	Good	Good granularity	Good	Good	Good	Excellent	FALSE
6	Good	Good	Too general	Good	Good	Good	Good	FALSE
	Fair	Fair	Good granularity	Fair	Excellent	Excellent	Good	FALSE
	Good	Fair	General	Good	Good	Good	Good	FALSE
7	Good	Good	Too general	Good	Good	Good	Good	FALSE
	Good	Fair	Good granularity	Excellent	Good	Good	Excellent	FALSE
	Good	Good	Good granularity	Good	Good	Good	Good	FALSE
8	Good	Good	Too general	Good	Good	Good	Good	FALSE
	Good	Good	Detailed	Good	Good	Good	Fair	FALSE
	Good	Good	Good granularity	Good	Good	Good	Good	FALSE
9	Good	Excellent	Detailed	Good	Excellent	Excellent	Good	FALSE
	Good	Excellent	Good granularity	Excellent	Excellent	Good	Good	FALSE
	Good	Excellent	Good granularity	Excellent	Excellent	Excellent	Excellent	FALSE
10	Good	Excellent	General	Good	Good	Excellent	Good	FALSE
	Very poor	Poor	Good granularity	Excellent	Very poor	Poor	Fair	FALSE
	Good	Fair	Good granularity	Excellent	Fair	Excellent	Excellent	FALSE
11	Good	Excellent	Good granularity	Good	Excellent	Excellent	Excellent	FALSE
	Good	Excellent	Good granularity	Excellent	Excellent	Excellent	Excellent	FALSE
	Good	Excellent	Good granularity	Excellent	Excellent	Good	Excellent	FALSE
12	Good	Good	General	Good	Good	Good	Good	FALSE
	Fair	Poor	Good granularity	Excellent	Good	Fair	Good	FALSE
	Very poor	Poor	Too general	Excellent	Very poor	Very poor	Good	FALSE
13	Excellent	Excellent	Good granularity	Excellent	Excellent	Excellent	Excellent	FALSE
	Good	Excellent	Good granularity	Excellent	Fair	Fair	Excellent	FALSE
	Good	Fair	General	Very poor	Excellent	Good	Good	FALSE
14	Good	Excellent	Good granularity	Good	Good	Good	Good	FALSE
	Good	Good	Detailed	Good	Good	Fair	Good	FALSE
	Good	Excellent	Detailed	Excellent	Excellent	Good	Good	FALSE
15	Good	Good	Good granularity	Good	Excellent	Excellent	Excellent	FALSE
	Good	Good	Detailed	Excellent	Excellent	Fair	Excellent	FALSE
	Good	Good	Detailed	Good	Excellent	Excellent	Excellent	FALSE
16	Good	Good	Good granularity	Good	Good	Good	Good	FALSE
	Good	Good	General	Excellent	Excellent	Fair	Good	FALSE
	Good	Good	General	Good	Good	Good	Good	FALSE
17	Good	Excellent	General	Good	Good	Good	Good	FALSE
	Poor	Poor	Detailed	Excellent	Fair	Poor	Good	FALSE
	Fair	Poor	General	Good	Fair	Poor	Good	FALSE
18	Good	Excellent	Good granularity	Good	Good	Good	Good	FALSE
	Excellent	Excellent	Good granularity	Excellent	Excellent	Excellent	Excellent	FALSE
	Excellent	Excellent	Good granularity	Good	Good	Good	Good	FALSE

Supplementary Table 3: Human evaluation results of abstract-based profiles.

Faculty	Overall impression	Factual Accuracy	Granularity	Readability	Comprehensiveness	Specificity	Conciseness	Identified as human
1	Good	Good	Detailed	Good	Excellent	Good	Fair	TRUE
	Good	Excellent	Too detailed	Fair	Fair	Poor	Excellent	FALSE
	Good	Excellent	Detailed	Good	Good	Fair	Fair	FALSE
2	Fair	Good	Too detailed	Good	Good	Fair	Very poor	FALSE
	Poor	Good	General	Poor	Poor	Very poor	Poor	FALSE
	Fair	Good	Detailed	Fair	Good	Fair	Poor	FALSE
3	Fair	Fair	Too detailed	Good	Fair	Poor	Very poor	FALSE
	Fair	Good	Detailed	Good	Fair	Fair	Poor	FALSE
	Poor	Fair	Too detailed	Excellent	Excellent	Very poor	Very poor	FALSE
4	Fair	Good	Detailed	Excellent	Excellent	Good	Good	FALSE
	Good	Good	Detailed	Good	Excellent	Good	Good	FALSE
	Fair	Good	Too detailed	Good	Good	Very poor	Good	FALSE
5	Fair	Fair	Too detailed	Poor	Good	Fair	Fair	FALSE
	Fair	Good	Too detailed	Fair	Fair	Fair	Fair	FALSE
	Fair	Good	General	Fair	Excellent	Fair	Fair	FALSE
6	Fair	Good	Too detailed	Fair	Fair	Fair	Fair	FALSE
	Good	Good	Too detailed	Poor	Fair	Poor	Fair	FALSE
	Fair	Fair	Detailed	Fair	Fair	Poor	Fair	FALSE
7	Poor	Fair	Too detailed	Poor	Poor	Poor	Poor	FALSE
	Fair	Fair	Too detailed	Fair	Fair	Fair	Fair	FALSE
	Fair	Fair	Detailed	Fair	Fair	Poor	Fair	FALSE
8	Poor	Fair	Too detailed	Poor	Fair	Poor	Poor	FALSE
	Fair	Fair	Too detailed	Fair	Fair	Fair	Fair	FALSE
	Fair	Poor	Too detailed	Poor	Fair	Poor	Poor	FALSE
9	Fair	Fair	Too detailed	Poor	Poor	Poor	Poor	FALSE
	Fair	Fair	Too detailed	Good	Excellent	Poor	Very poor	FALSE
	Fair	Good	Detailed	Fair	Fair	Fair	Poor	FALSE
10	Poor	Fair	Too detailed	Poor	Poor	Poor	Poor	FALSE
	Poor	Good	Detailed	Good	Very poor	Very poor	Very poor	FALSE
	Fair	Poor	General	Poor	Poor	Fair	Fair	FALSE
11	Poor	Fair	Too detailed	Poor	Poor	Poor	Poor	FALSE
	Poor	Fair	Too detailed	Fair	Excellent	Poor	Poor	FALSE
	Poor	Fair	Too detailed	Good	Excellent	Poor	Very poor	FALSE
12	Fair	Good	Too detailed	Poor	Poor	Poor	Poor	FALSE
	Good	Good	Detailed	Fair	Fair	Fair	Fair	FALSE
	Very poor	Excellent	Too detailed	Excellent	Excellent	Poor	Poor	FALSE
13	Poor	Poor	Too detailed	Poor	Poor	Poor	Poor	FALSE
	Fair	Fair	Detailed	Fair	Fair	Fair	Fair	FALSE
	Very poor	Poor	Too detailed	Good	Excellent	Very poor	Very poor	FALSE
14	Poor	Poor	Too detailed	Poor	Poor	Poor	Poor	FALSE
	Fair	Good	Too detailed	Fair	Fair	Poor	Poor	FALSE
	Fair	Fair	Too detailed	Poor	Good	Very poor	Very poor	FALSE
15	Poor	Poor	Too detailed	Poor	Poor	Poor	Poor	FALSE
	Poor	Poor	Too detailed	Very poor	Excellent	Poor	Fair	FALSE
	Fair	Poor	Too detailed	Very poor	Fair	Poor	Very poor	FALSE
16	Poor	Fair	Too detailed	Poor	Poor	Poor	Poor	FALSE
	Poor	Fair	Too detailed	Excellent	Good	Poor	Poor	FALSE
	Fair	Fair	Detailed	Good	Poor	Fair	Poor	FALSE
17	Poor	Fair	Too detailed	Poor	Poor	Poor	Poor	FALSE
	Poor	Fair	Too detailed	Excellent	Good	Poor	Fair	FALSE
	Poor	Fair	Too detailed	Poor	Fair	Poor	Poor	FALSE
18	Poor	Fair	Too detailed	Poor	Poor	Poor	Poor	FALSE
	Poor	Fair	Too detailed	Excellent	Excellent	Poor	Poor	FALSE
	Poor	Good	Too detailed	Good	Excellent	Fair	Fair	FALSE

Supplementary Table 4: Unique MeSH Terms used by human-written profiles.

academia	endoderm	islam
air	endophenotypes	israel
air pollution	eukaryotic cells	italy
anatomy	exome	jaw
anthropology	exome sequencing	jews
aplysia	extracellular matrix	kansas
archaea	extreme environments	laboratories
arizona	faculty	leg
art	flaviviridae	leucine zippers
arthritis	focus groups	lewy bodies
autoimmunity	foundations	linkage disequilibrium
beijing	freedom	literature
biophysics	friends	logic
biostatistics	fruit	mammals
biotechnology	gastroenterology	massachusetts
birth order	gastrulation	mathematics
blood volume	genetic linkage	meaningful use
books	genetic techniques	medical oncology
boston	genomic medicine	melanogenesis
california	germany	mesoderm
cations	gestures	microbiology
cellular reprogramming	glycosylation	microphysiological systems
chart	government	micrornas
chicago	growth cones	morbidity
chloroquine	gynecology	moscow
cisplatin	habits	muscle relaxation
cocaine	hair diseases	muscle strength
cognitive psychology	hallucinations	mutagenesis
cognitive science	health policy	mutagens
common cold	hearing	myocarditis
comprehension	hematology	nanofibers
computer systems	hiv antibodies	necrosis
computers	hope	nephrology
congress	hospital medicine	neural prostheses
conjunctivitis	hospitals	neural tube
coronaviridae	humanities	neuroendocrine cells
crohn disease	hypertriglyceridemia	neurology
culture	hypnosis	news
demography	iga vasculitis	north carolina
desmosomes	implementation science	notochord
digital health	inositol	nursing research
dna breaks	intention	obstetrics
dna cleavage	internal medicine	optic chiasm
document analysis	inventors	optical imaging
editorial	iontophoresis	organ size

organogenesis
osteocalcin
outline
paper
parahippocampal gyrus
paralysis
parasites
pennsylvania
photons
physicians
poliomyelitis
politics
postdoctoral training
private sector
probability
problem solving
projection
protein binding
psychophysics
pubmed
quantum dots
reflex
relaxation
reperfusion
reperfusion injury
review
rosiglitazone
san francisco
sarcoma
school nursing
serum
sign language
skeleton
social networking
somatosensory cortex
sound
spatial learning
spatial memory
students
surgeons
survival analysis
synaptic vesicles
texas
therapeutics
translating
triglycerides
united states
universities
urothelium
utah
vasculitis
virginia
virus replication
visual pathways
washington
wound healing
zinc
